# Targeting CEA in metastatic triple negative breast cancer with image-guided radiation followed by Fab-mediated chimeric antigen receptor (CAR) T-cell therapy

**DOI:** 10.3389/fimmu.2024.1499471

**Published:** 2024-12-20

**Authors:** Eric Aniogo, Maciej Kujawski, Dennis Awuah, Seung E. Cha, Ruby Espinosa, Susanta Hui, Hemendra Ghimire, Paul J. Yazaki, Christine E. Brown, Xiuli Wang, John E. Shively

**Affiliations:** ^1^ Department of Immunology and Theranostics, City of Hope, Duarte, CA, United States; ^2^ T-Cell Therapeutic Laboratory, City of Hope, Duarte, CA, United States; ^3^ Department of Radiation Oncology, City of Hope, Duarte, CA, United States; ^4^ Department of Hematology & Hematopoietic Cell Transplantation, City of Hope Beckman Research Institute and Medical Center, Duarte, CA, United States

**Keywords:** chimeric antigen receptor T cells, triple-negative breast cancer, carcinoembryonic antigen, image-guided radiation therapy, cell immunotherapy

## Abstract

**Introduction:**

Although CAR-T cell therapy has limited efficacy against solid tumors, it has been hypothesized that prior treatment with Image-Guided Radiation Therapy (IGRT) would increase CAR-T cell tumor infiltration, leading to improved antigen specific expansion of CAR-T cells.

**Methods:**

To test this hypothesis in a metastatic triple negative breast cancer (TNBC) model, we engineered two anti-CEA single-chain Fab (scFab) CAR-T cells with signaling domains from CD28zeta and 4-1BBzeta, and tested them *in vitro* and *in vivo*.

**Results:**

The anti-CEA scFab CAR-T cells generated from three different human donors demonstrated robust *in vitro* expression, expansion, and lysis of only CEA-positive TNBC cells, with the CD28z-CAR-T cells showing the highest cytotoxicity. IFN-γ and granzyme B release assays revealed significantly higher IFN-γ production at a 4:1 effector-to-target (E:T) ratio in CD28z-CAR-T cells compared to 4-1BBz-CAR-T cells. Treatment of CEA-positive TNBC MDA-MB231 xenografts in the mammary fat pads of NSG mice, that produced spontaneous lung metastases over time, resulted in significant tumor growth reduction compared to either therapy alone (p<0.01). Immunohistochemical (IHC) analysis revealed that only combined IGRT and CAR-T therapy resulted in the elimination of lung metastases.

**Discussion:**

These findings demonstrate that the combination of IGRT and anti-CEA scFab CAR-T therapy induces a strong antitumor response, effectively targeting both the primary tumor and distant metastatic lesions in the lungs, thus demonstrating that IGRT enhances CAR-T cell infiltration, persistence, and overall efficacy within both primary and metastatic lesions.

## Introduction

Breast cancer, that originates from the epithelial cells of the breast ducts and lobules, is the most common cancer affecting women. It is estimated that women in developed countries have about 1 in 8 chance of developing breast cancer in their lifetime (1). Triple-negative breast cancer (TNBC) is an aggressive and metastatic form that accounts for 15–20% of all breast cancers (2). TNBC cells lack estrogen receptor (ER), progesterone receptor (PR), and the human epidermal growth factor receptor 2 (HER2), making them very difficult to treat and contributing to their poor prognosis (3). Current breast cancer treatments include surgery, chemotherapy, and radiotherapy but these methods are often associated with adverse effects such as cosmetic damage, pancytopenia, nausea, diarrhea among others (4). Over the years, immunotherapy has gained approval for breast cancer treatment, with vaccines and immune checkpoint blockade being employed (5). Thus far, adoptive cell therapy, including chimeric antigen receptor (CAR) T cell immunotherapy has shown clinical promise in hematological malignancies such as leukemia, lymphoma, and multiple myeloma (6).

CAR-T cell immunotherapy involves engineering a patient’s T cells to express a chimeric antibody-T cell receptor, thereby redirecting them for effective tumor targeting without the need for MHC (7). However, the clinical efficacy of CAR-T cells for most solid tumors is substantially limited due to insufficient trafficking, poor functional persistence and inhibition by the immunosuppressive tumor microenvironment (8). To address these challenges, CAR-T cell therapy has been combined with other clinically approved treatments, including radiotherapy (9).

Image-guided radiation therapy (IGRT) is an approved therapeutic procedure used in combination regimens for managing various malignancies (10, 11). This procedure alters the tumor microenvironment (TME) by disrupting its mechanical and functional barriers, leading to the release of proinflammatory cytokines that can activate systemic immunomodulatory effects beneficial for CAR-T cells (12). Recent immunotherapy strategies have combined radiation with immune checkpoint inhibitors to enhance immune responses against solid tumors (13, 14). Targeted radiotherapy also causes the release of death-associated molecular patterns (DAMPs), that stimulate immune system activation, including vascular remodeling, neoantigen expression, and endothelial cellular adhesion changes that promote immune cell infiltration into the tumor (15). Although CAR-T cell and IGRT therapies have limitations as single-agent treatments for solid tumors, IGRT targets the TME of imageable tumors but misses micromets while CAR-T cells can reach both primary tumors, and micrometastases but are often ineffective due to immunosuppressive TME (16, 17). Hence their combination can create a synergistic effect that enhances therapeutic efficacy.

Carcinoembryonic antigen (CEA or CEACAM5) is an oncodevelopmental cell surface glycoprotein identified with the Cluster of Differentiation designation CD66e (18). It is a tumor-associated protein that is highly expressed in various solid tumors, including colon, gastrointestinal, breast, and lungs (19). CEA plays a significant role in tumor detection, prognosis, treatment monitoring, and its upregulation associated with the progression, proliferation and migration of metastatic breast tumors (20). Since most anti-CEA antibodies cross-react with other members of the CEA family found in normal tissues, the use of a CEA specific antibody such as our humanized M5A antibody is critical (21). Our previous study in immunocompetent CEA transgenic (CEA-Tg) mice that express the same antigen as human in endogenous organs demonstrated specific CAR-T cell responses in CEA positive breast and colon tumor models without any observed off-target effects (22). Similarly, Chmielewski and colleagues used anti-CEA CAR-T therapy in CEA-Tg mice bearing pancreatic tumors with no evidence of destruction of CEA-positive normal tissues (23).

Currently, most CAR-T constructs utilize a single-chain variable fragment (scFv) antigen-binding domain that have a tendency to aggregate and reduce the antigen-antibody interaction (24). This aggregation can result in the formation of CAR aggregates on the surface of CAR-T cells, leading to unexpected signaling and constitutive activation of T cells through an antigen-independent mechanism known as tonic signaling. This phenomenon contributes to faster signal loss and reduced efficacy of CAR-T therapy (25). Alternatives to scFv-based CARs, such as Fab-based chimeric antigen receptor T targeting CD276 (26) and CD19 (27, 28), have been explored and tested *in vitro* as substitutes for scFv CAR-T cells.

In this study, we assessed the specificity and functional killing efficacy of scFab anti-CEA CAR-T cells against CEA-positive TNBC cells both *in vitro* and in a xenograft metastatic tumor model. We hypothesized that antigen derived expansion of CAR Ts would occur at both the primary and metastatic sites and benefit from IGRT directed only at the primary site.

## Materials and methods

### Generation of CAR-specific T cells

**Figure 1:**
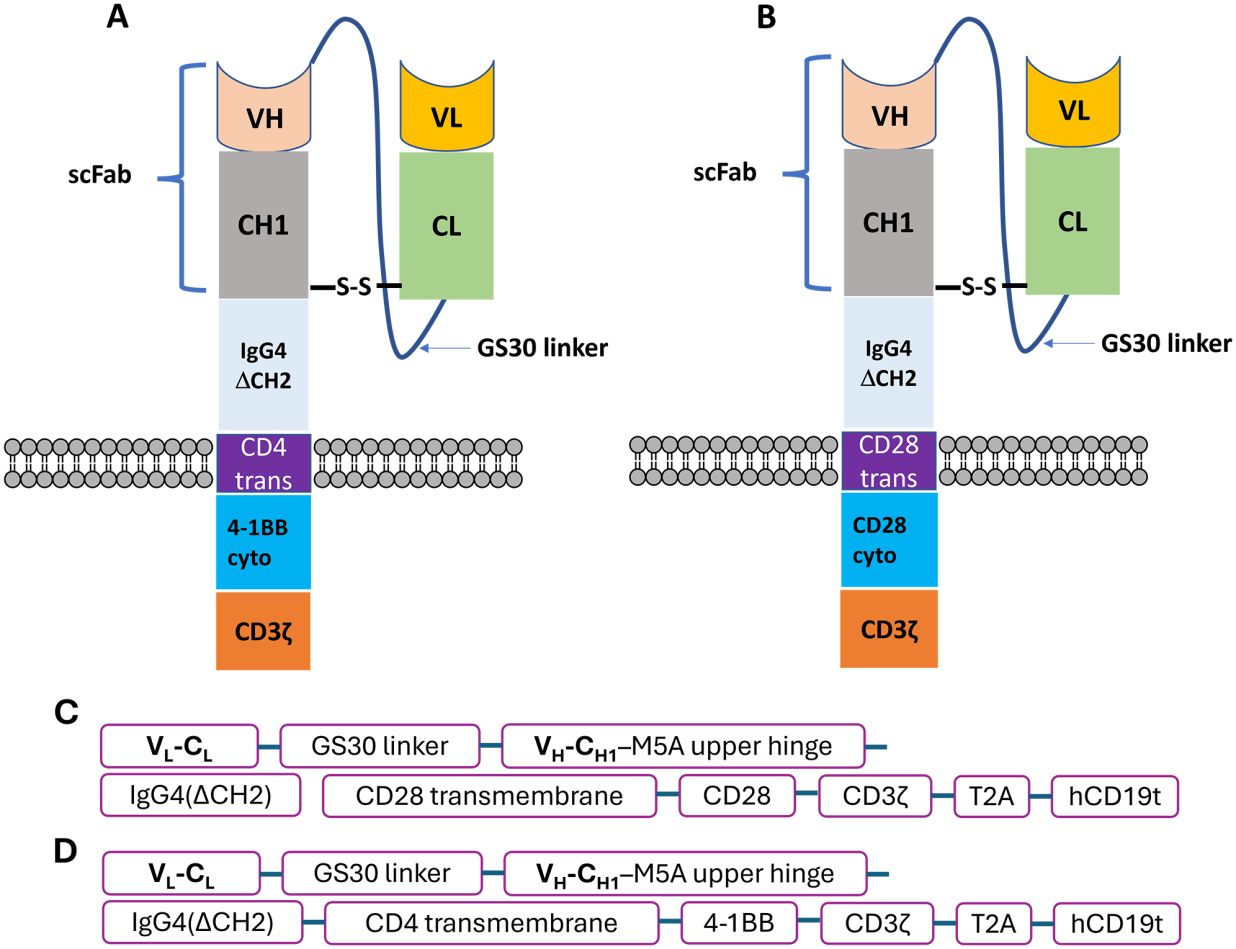
Schematics of humanized anti-CEA (M5A) scFab-CAR-T. **(A)** 4-1BB and **(B)** CD28 CAR constructs. Two constructs were tested, with cytoplasmic domain corresponding to second generation CAR-Ts. **(C, D)** The two scFab CAR-T constructs have distinct signaling domains; CD28-CD3zeta and 4-1BB-CDzeta configuration, both co-expressing hCD19t to allow identification of expressed CARs.

The M5A-targeted 28z CAR construct consists of an hM5A(Fab) domain linked to an IgG4 hinge region with CD28 transmembrane and co-stimulatory domains, and the intracellular signaling domain of CD3ζ (**Figure 1A**). In contrast, the 4-1BBz CAR construct includes an hM5A(Fab)-IgG4-derived Fab linked to the transmembrane domain of CD4, the intracellular domain of 4-1BBz, and the CD3ζ intracellular signaling domain (**Figure 1B**). The two scFab CAR-T plasmids, driven by the EF1p promoter, feature distinct signaling domains; one with a CD28-CD3zeta configuration and the other with a 4-1BB-CD3zeta configuration. Both constructs include a T2A ribosomal skip sequence that separates the codon-optimized CAR sequence from the truncated human CD19t (hCD19t) to allow for identification and enrichment of expressed CARs. (**Figures 1C, D**). Leukapheresis products were obtained from healthy donors under COH-approved protocols. Peripheral blood mononuclear cells (PBMCs) were separated using density gradient centrifugation on Ficoll-Paque and depleted of CD14+ and CD25+ cells. T naïve/memory cells were then selected from the resulting negative fraction using CD62L+ magnetic beads and activated with CD3/CD28 beads. Activated cells were transduced with the different CAR lentiviral vectors and expanded as previously described (22). Similarly, the lentiviral plasmid construct used for dual transduction of luciferase CAR-T cells was generated using the epHIV7 vector, with luciferase expression driven by the EF1α promoter.

### Antibodies and flow cytometry

Flow cytometry was performed for the phenotypic analysis of T cells and/or tumors. Briefly, CAR-T cells (1 × 10^5^) were suspended in FACS staining solution and incubated with fluorescently labeled antibodies for 15 min at 4°C. Unless otherwise stated, cells were stained with mouse anti-human CD3, CD8, CD4, PD-1, Tim3 and CD19 antibodies (BD Biosciences). Anti-CD45RA and anti-CD62L antibodies (BD Biosciences) were used to assess the differentiation status of CAR-T cells.

### Effector: target cell killing assay

Mock or anti-CEA CAR-T cells were incubated with either WT-MDA-MB231 (ATCC; HTB-26) or MDA-MB231 transfected with CEA and GFP (29) at varying effector to target (E:T) ratios (0.5:1, 1:1, 2:1, 4:1). Briefly, 100 µl of target cells in fluoroBrite DMEM complete medium (Gibco, A18967-01) was first seeded in 96-well plates, followed by addition of 100 µl of effector cells to the same well. The mixture was incubated overnight at 37°C for 24 hrs. Target-only cells were used as controls. The percentage cell cytotoxicity was calculated following the formula shown below, where 100% cell viability (value_max_) was measured by averaging the fluorescence readings of the target cells without any T cells. The fluorescence for each well co-cultured (target and effector) is labeled as the experimental value (value_exp_). The background was subtracted as (value_min_) from both value_max_ and value_exp_. The fraction of live cell fluorescence was calculated by dividing ( $value_{\exp}-value_{\min}$ ) by ( $value_{\max}-value_{\min}$ ). To determine the fluorescence loss due to GFP-expressing target cell death, this fraction was subtracted from 1. The resulting value was then multiplied by 100% to obtain the percentage of cell cytotoxicity.


$$\%\;cell\;cytotoxicity=\left(1-\;\frac{value_{exp}-\;value_{min}}{value_{max}-\;value_{min}}\right)\;\times 100\%$$

### Cytokine measurement

Cytokine release was analyzed using an ELISA kit assay (BioLegend; 430104 and R&D systems; DY2906-05, for human IFN-γ and granzyme B respectively) according to the manufacturer’s instructions. In brief, 50 µL of supernatant from the co-culture killing assay was collected and diluted 1:10 with the kit buffer solution. Cytokine levels for IFN-γ and granzyme B were measured using a human ELISA kit, with absorption readings in triplicates taken on a CLARIOstar instrument.

### Measurement of cellular degranulation and T-cell exhaustion

CD107a was used to assess the level of cellular degranulation, a prerequisite for T-cell mediated cytolysis. Briefly, target cells were co-cultured with anti-CEA CAR-T cells for 6 hours at an effector-to-target (E:T) ratio of 2:1, with 0.26% w/w of Golgi stop (BD; 554724) added to the media. After the incubation period, the CAR-T cells were collected and stained with 1:20 dilutions of anti-CD3, CD4, CD8, and CD107a antibodies for 25 minutes on ice. The cells were then washed and analyzed using a flow cytometer.

To measure T cell exhaustion, target cells and scFab CAR-T cells were co-cultured at an effector-to-target (E:T) ratio 2:1 for three days. After the incubation period, the cells were washed with 1% PBS and subsequently stained with PD-1 and TIM-3 markers.

### Animal study

All animal studies were performed with NOD/SCID/IL-2rg (NSG; 6 – 10 weeks old; Jackson Laboratory; Jax 005557) (IACUC 91037). Animals were housed in pie cages, in a pathogen free room with a maximum of 5 mice per cage. On Day 1, mice were engrafted orthotopically with 5x10^5^ MB231/CEA-Luciferase positive cancer cells in 50 μL of PBS and Matrigel solution at 1:1 ratio (Corning, 356237) into the mammary fat pat of the female mice using 28G Insulin Syringes (BD, 329461). MB231/CEA-Luciferase positive cells were used to monitor tumor metastases using anti-firefly luciferase. Tumor size was measured and established tumors (50-75 mm^3^) on day 9 were randomly assigned to groups (n=4-5 mice per group). On day 10 post tumor engraftment, both radiations only group and combination treatment groups received 10 Gy of irradiation each. On day 11, combination group and CAR-T groups only were treated with 1x10^6^ scFab anti-CEA CAR-T cells in 200 μL PBS were injected intravenously. Untreated groups received PBS. Tumor growths were monitored and measured with caliper and growth endpoint were set at >1500 mm^3^.

To track CAR-T cell tumor infiltration, activated human T-cells were co-transduced with lentivirus encoding GFP-firefly luciferase and scFab CAR-T. The double-positive cells were then isolated using a flow cytometry cell sorter. For the experiment, NSG mice bearing MDA-MB-231/CEA tumors (which do not express luciferase) were utilized and followed the same experimental procedure described above. On day 6, the mice were divided into treatment groups: untreated controls, CAR-T cells alone, fractionated radiation (4x 2.5 Gy), or single-dose IGRT (10 Gy). The untreated control group included 2 mice, while the other groups consisted of 3-5 mice each. The fractionated radiation group received 2.5 Gy daily from day 7 to day 10, while the single-dose group was treated with 10 Gy once on day 10. On day 11, both the fractionated and single-dose groups received an intravenous injection of 1x10^6^ anti-scFab-CEA CAR-T cells expressing luciferase in 200 μL PBS. Untreated mice served as controls. Mice were imaged weekly using the LAGO bioluminescence imaging system (Spectral Instruments Imaging, LLC, Tucson, USA), and luciferase signals were quantified for comparative analysis.

### Tissue collection and analysis

At endpoint, lungs tissue and tumors were collected in cold PBS. For flow cytometry analysis, small fractions of tumor were cut into pieces and digested with Tumor Dissociation Kit, Mouse (MACS, 130-096-730) and gentleMACS C Tubes (MACS, 130-096-334), as per manufacturer’s directions. The cells were meshed on a 0.40 μM cell strainer (Corning, 431750) and lysed with Red Blood Cell Lysis Buffer Hybri-Max (Sigma, R7757). Thereafter, the cells were stained with fluorescent antibodies for flow cytometry. To measure IFN-γ production, cells were re-stimulated with Cell Activation Cocktail (with Brefeldin A; Biolegend, 423303) in 10% FBS RPMI media for 4 hours at 37°C. Following, cells were stained for surface markers (CD4, CD8, PD-1) and viability marker (Zombie UV, BioLegend). These cells were fixed and permeabilized with Fixation/Permeabilization kit (ThermoFisher) according to the manufacture’s protocol and stained for intracellular IFN-γ (BioLegend) and analyzed by flow cytometry.

For immunohistochemistry (IHC), the harvested tumor and lungs tissues were fixed with 4% paraformaldehyde for 3 days and thereafter stored in 70% ethanol. Tissues were washed in PBS and frozen on dry ice using O.C.T. (Fisher HealthCare, 4585) in vinyl Specimen molds (Sakura, 4557) for H&E and IHC staining. Tissue block slides were stained with anti-firefly (luciferase detection) and mouse anti-human CD3 antibodies.

### Statistical analysis

The results were analyzed using Prism statistical software using the T-test, one way ANOVA, or two-way ANOVA to compare the two experimental groups. Statistical analysis of more than three groups will be based on two-way ANOVA and Sidak’s multiple comparison test. A threshold of *p* < 0.05 was considered statistically significant throughout. * *p* < 0.05, ** *p* < 0.01, ***p < 0.001, ****p < 0.0001.

## Results

### Production of scFab-CAR T cells

**Figure 2:**
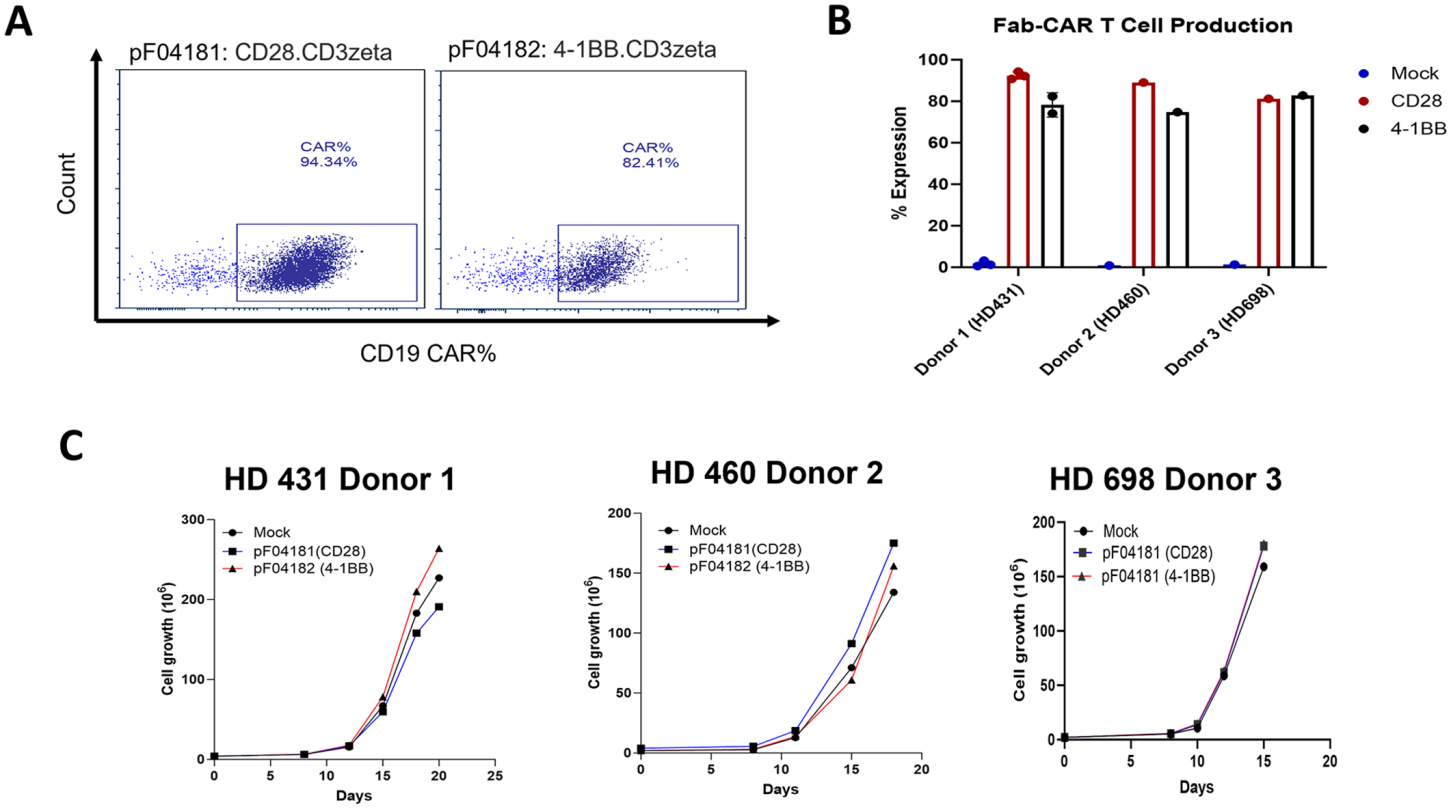
scFab-CAR-T production and expression on T cells. **(A)** Transduction efficiency was assessed by flow cytometry for CD19t expression of transduced T cells following enrichment with anti-CD19 beads. **(B)** Percentage expression of scFab-CAR-T cell production across T cells from three different donors. **(C)** Growth curves showing the expansion of scFab-CAR-T from three donors’ post-transduction.

Our initial studies used an all murine anti-CEA scFv CAR T derived from the anti-CEA monoclonal antibody T84.66 in immunocompetent CEA Tg mice (22). However, a scFv CAR-T derived from the humanized version of this antibody (21) exhibited poor stability in T cells (data not shown). Consequently, we designed and tested anti-CEA scFab CAR-T cells, that included constant and variable domains from the heavy and light chains of the humanized anti-CEA antibody (M5A) plus signaling domains CD28zeta or 4-1BBzeta (**Figure 1**). The construct also included an expression cassette for a truncated human CD19 gene (hCD19t) to allowing enrichment of transfected from non-transfected T cells (30). CD3+ T naïve/memory cells (**Supplementary Figure S1**) from human donors were transduced with or without (mock – untransduced) lentivirus encoding the anti-CEA scFab, and transduction efficiency was confirmed by flow cytometry monitoring hCD19t expression following enrichment with anti-CD19 beads (**Figure 2A**). CAR-T cells produced from three different human donors (HD) were enriched using anti-CD19 magnetic beads that resulted in final transduction efficiencies of 94%, 89% and 81% for CD28z-CAR-T and 82%, 75%, and 83% for 4-1BBz-CAR-T cells, respectively (**Figure 2B**). The mock transfected T cells showed no CAR-T cell expression. The growth expansion curve of T cells post-CAR-T production from three different donors is shown (**Figure 2C**), demonstrating good activation and proliferation of CAR-T cells. These results indicate that using the scFab fragment of the humanized M5A antibody for CAR-T production does not negatively impact the activity of the transduced human T cells.

### Target specificity of scFab anti-CEA CAR-T cells

**Figure 3:**
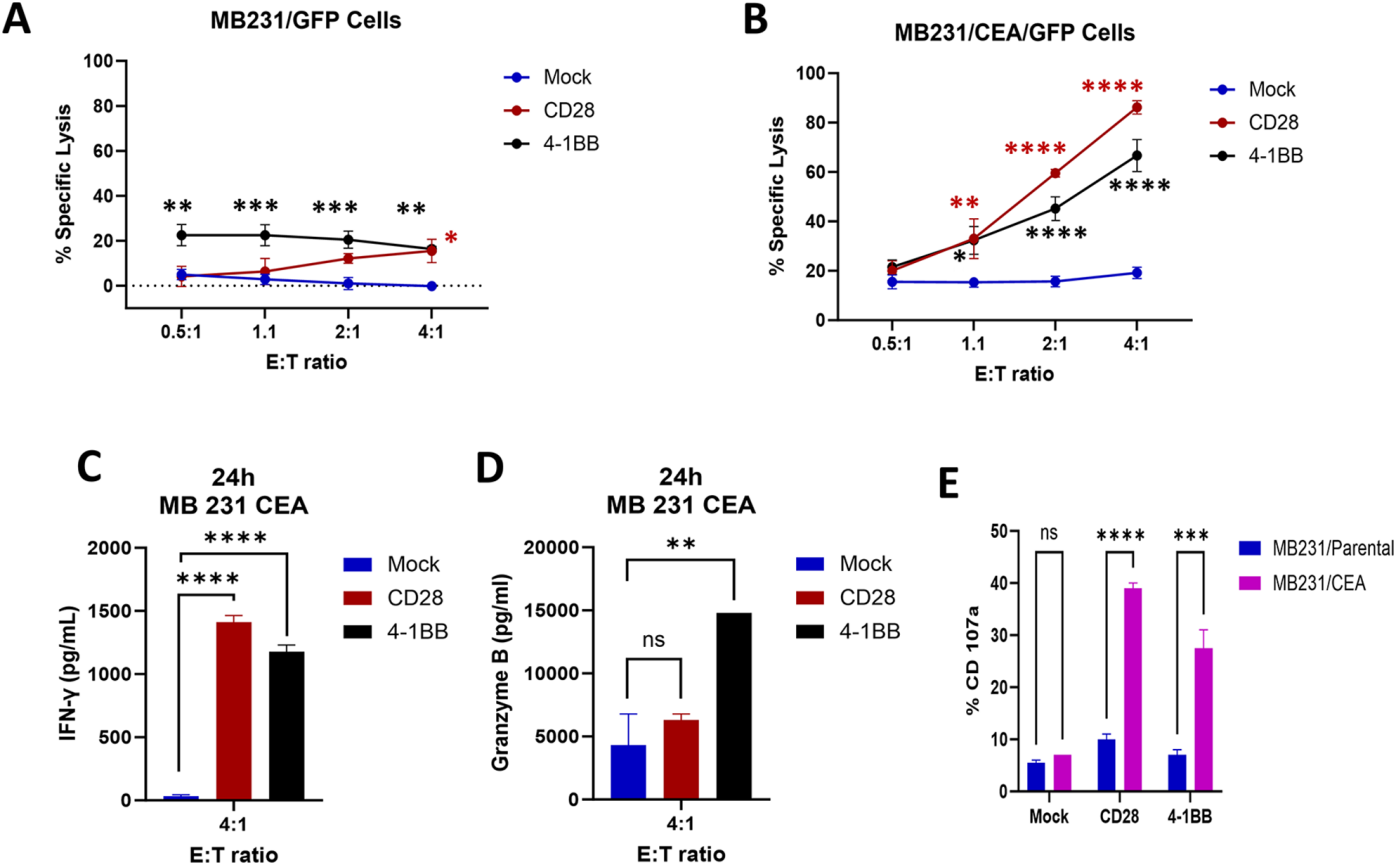
*In Vitro* scFab-CAR T-cell cytotoxic assays; **(A)** Cytotoxicity against CEA-negative MDA-MB231 parental cells. **(B)** Cytotoxicity against CEA-positive MDA-MB231 cells. Both versions of scFab CAR-T cells exhibit antigen-specific lysis of target cells. **(C, D)** Analysis of IFN-γ and granzyme B production by mock and two versions of scFab CAR-T cells against CEA-positive MDA-MB231 cells. **(E)** Expression of the CD107a degranulation marker in both CAR-T cells when co-incubated with CEA-positive and negative MDA-MB231 target cells. (Statistical analysis was performed using two-way ANOVA and Sidak’s multiple comparison test *p < 0.05, **p < 0.01, ***p < 0.001, ****p < 0.0001, ns - not significant).

To determine the antigen specificity of anti-CEA scFab CAR-T cells, CD28z-CAR-T, 4-1BBz-CAR-T, or mock T cells were incubated with triple-negative human breast cancer cells (MB231) transfected with CEA and GFP or GFP only (**Supplementary Figure S1**). These cells were used in increasing Effector: Target (E:T) ratios with CEA or GFP-only cells as positive controls for specific and nonspecific targeting, respectively. The specific lysis of CEA^+^ versus CEA^-^ target cells by scFab CAR-T cells was demonstrated, with the highest killing observed in CD28z-CAR-T cells. In contrast, the mock T cells were ineffective in killing the targets (**Figures 3A, B**). Measurement of IFN-γ and granzyme B by ELISA in the supernatants from the co-culture killing assay with CEA^+^ target cells showed an increased release of IFN-γ at 4:1 E:T ratio for CD28z-CAR-T cells (**Figure 3C**, **Supplementary Figure S2**). Conversely, 4-1BBz-CAR-T exhibited increased release of granzyme B (**Figure 3D**, **Supplementary Figure S2**). For control target cells, no IFN-γ or granzyme B was detected. In a separate experiment, the co-culture killing assay was extended for three days, and the expression levels of PD-1 and TIM-3 exhaustion markers were measured. The CD4 and CD8 T cell subpopulations in both CEA^-^ and CEA^+^ MDA-MB231 cells co-cultures showed increased expression of PD-1 but not Tim3 exhaustion markers on the effector cells (**Supplementary Figure S2**). The expression of the PD-1 marker was higher in CD28z-CAR-T cells compared to 4-1BBz-CAR-T cells. Furthermore, the expression of CD107a degranulation marker was higher in scFab CD28z-CAR-T cells than in 41BBz-CAR-T cells (**Figure 3E**). Together, these data confirm that both formats of scFab anti-CEA CAR-T cells specifically targeted CEA-expressing cells through antigen recognition and T cell activation.

### Image guided radiation therapy enhances the effectiveness of anti-CEA scFab CD28z-CAR-T cell therapy

**Figure 4:**
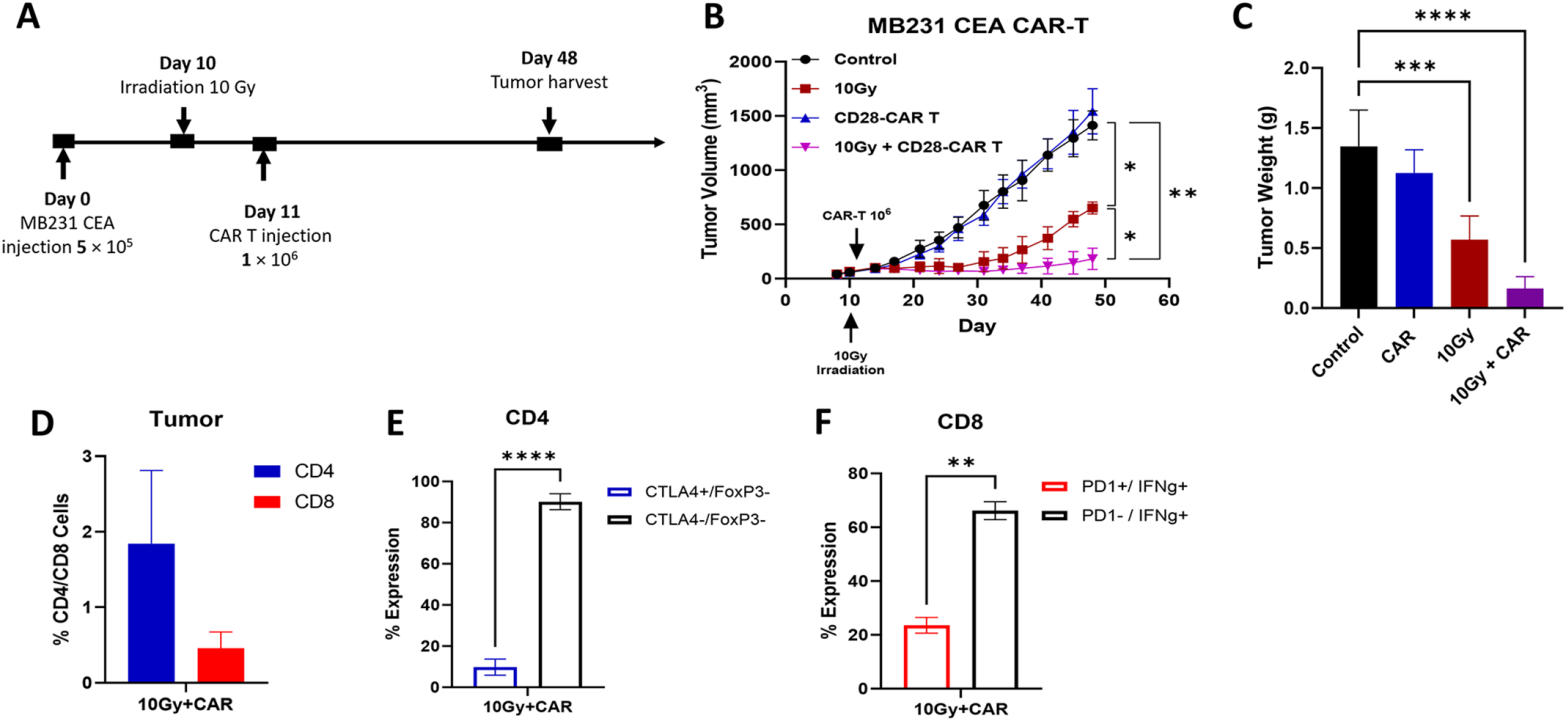
*In Vivo* therapeutic Efficacy of Anti-CEA scFab-CD28-CAR-T in combination with 10 Gy of IGRT. **(A)** Experimental design: orthotopic MDA-MB-231CEA-Luc positive tumors implanted in NSG mice were treated with 10 Gy IGRT on day 10, followed by an intravenous injection of 1 × 10^6^ anti-CEA scFab CAR-T cells on day 11. Tumor size was monitored with calipers until reaching 1500 mm³ (for controls and CAR T groups). **(B)** MDA-MB-231CEA-Luc tumor growth curves (n=4-5 mice per group). The combination therapy of 10 Gy IGRT and scFab-CAR-T cells was statistically significant (p<0.01). **(C)** Tumor weight measurements after euthanasia on day 48 showed a significant difference (p<0.0001) in tumor weight between the control and combined treatment groups. **(D)** Tumor analysis for T cell subpopulations (CD4/CD8) using flow cytometry and their corresponding expression of exhaustion markers in CD4 **(E)** and CD8 **(F)** subpopulations. (Statistical analysis was performed using two-way ANOVA and Sidak’s multiple comparison test *p < 0.05, **p < 0.01, ***p < 0.001, ****p < 0.0001).

To evaluate the therapeutic efficacy of anti-CEA scFab CAR-T cells against solid tumors, MB231/CEA breast tumors were orthotopically implanted in the mammary fat pads of immunocompromised NSG mice. MDA-MB231 xenografts were chosen due to their propensity to spontaneously form lung metastases (31). Established tumors in mice were treated with a 10 Gy single dose of IGRT alone, scFab CAR-T alone, or IGRT followed by scFab CAR T the following day (**Figure 4A**). A significant tumor growth reduction was observed in the mice treated with the combination of IGRT and anti-CEA scFab CAR-T cells compared to either monotherapy. The combination therapy was statistically significant (p<0.01) compared to scFab CD28z-CAR-T cells alone and untreated control (**Figure 4B**). The 10 Gy IGRT-treated mice showed significantly reduced tumor growth until day 30, after which the tumor growth escaped. On day 48, when the tumors in the control and scFab CAR-T cell-only treated mice reached maximum volume, the tumors and lungs from all mice were collected. Tumor weight measurements post-euthanasia showed a significant difference (p<0.0001) between the control and combined treatment groups (**Figure 4C**). At this endpoint, portions of the tumors were enzymatically digested and analyzed by flow cytometry for T cell phenotyping. Flow cytometry analysis revealed the presence of anti-CEA CAR-T cells in combination group only, and an increased CD4 T cell subpopulation compared to CD8 (**Figure 4D**). To assess T cell functions such as IFN-γ production and Treg activity, harvested T cells were restimulated for 4 hrs, followed by intracellular staining for IFN-γ and FoxP3. The analysis showed fewer exhausted cells stained with CTLA-4 in CD4 and PD-1 in the CD8 cell population. Additionally, there was an increased number of IFN-γ producing CD8 cells, that likely contributed to the improved anti-tumor activity and tumor inhibition (**Figures 4E, F**). The expression of PD-1 on CD4+ CAR T cells was only 20% with less than 1% of IFN-γ (data not shown). Repeating the experiment with a different donor T-cell, including mock and 4-1BB variants of the anti-CEA scFab CAR-T cells, also showed similar results with the best tumor inhibition observed in CD28-scFab-CAR-T (**Supplementary Figure S3**).

### IGRT improves the infiltration scFab anti-CEA CAR-T cells in solid tumors

**Figure 5:**
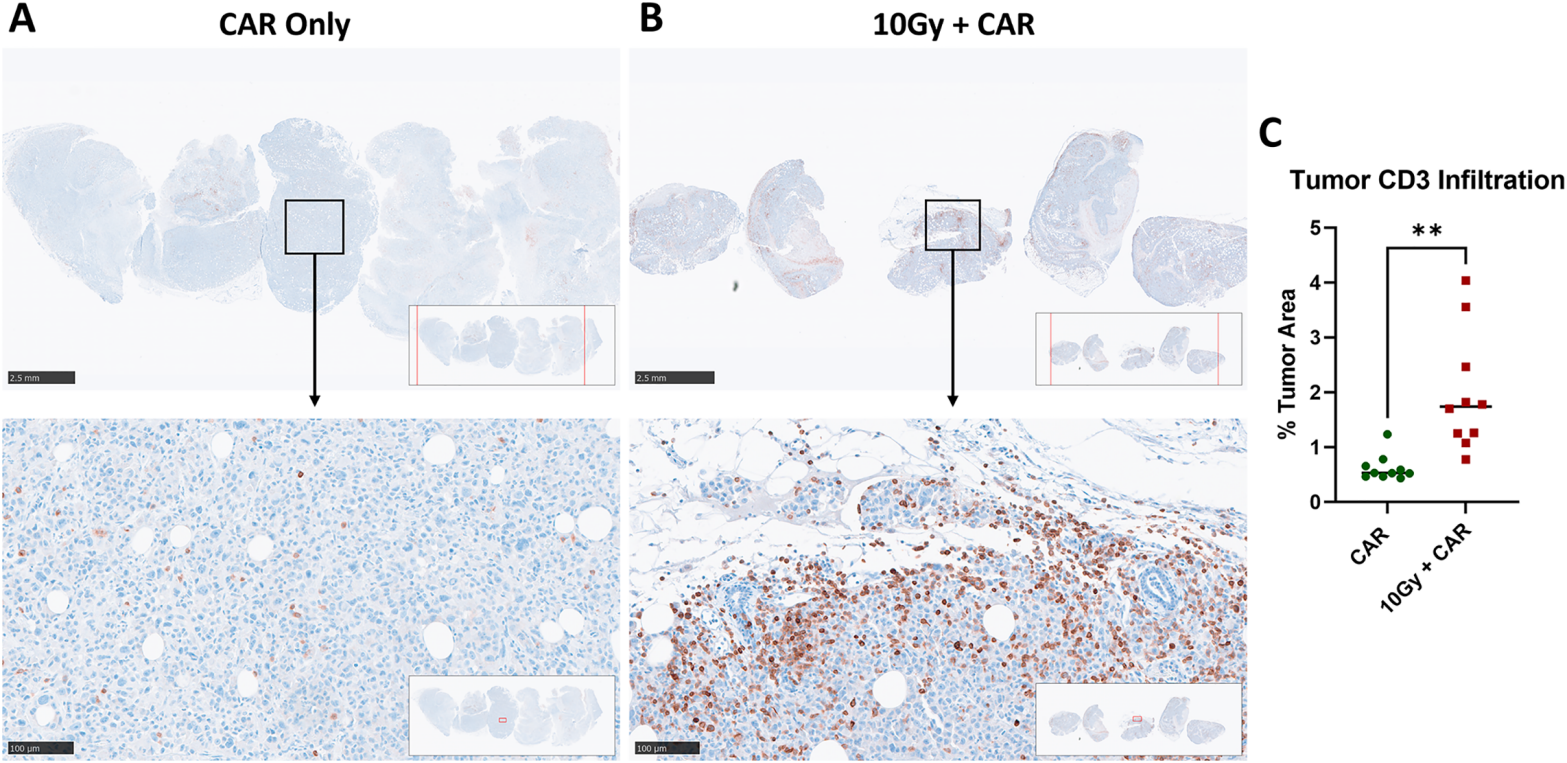
Comparative immunohistochemistry analysis of infiltrating anti-CEA CAR-T cells in MDA-MB231CEA-Luciferase Tumors with or without IGRT. NSG mice with orthotopic MDA-MB-231CEA-Luciferase-positive tumors were analyzed as **(A)** Tumors treated with CAR-T cells only and **(B)** Tumors receiving combination treatment of 10Gy IGRT and anti-CEA CAR-T cells. Tumors were monitored until they reached terminal size or until day 48, and then analyzed using immunohistochemistry. Anti-CD3 staining indicates infiltrating anti-CEA CAR-T cells, that were significantly increased in the combination therapy group. **(C)** Quantification of infiltrating CD3-positive T cells in MDA-MB-231CEA-Luciferase tumors (2 regions per tumor, 5 tumors each group) showed a significant increase in the combination treatment group compared to the anti-CEA CAR-T cell-only group (p < 0.01). (Statistical analysis was performed using student t-test **p < 0.01).

Anti-CD3 immunohistochemistry (IHC) analysis of the tumor tissues revealed poor infiltration of anti-CEA scFab CAR-T cells in the CAR-T only treated group (**Figure 5A**) compared to their significant infiltration in the combined therapy group (**Figure 5B**). CD3+ cells were abundant in the combination group even 37 days post a single injection of scFab anti-CEA CAR-T cells. Quantification of the infiltrating scFab CAR-T cells in the tumor showed a significant increase in the combination treatment group (**Figure 5C**). These findings suggest that IGRT facilitated the infiltration, persistence, and antitumor response of scFab CAR-T cells within solid tumor tissues.

### Combination IGRT with anti-CEA scFab CAR-T therapy prevents breast cancer metastasis to the lungs

**Figure 6:**
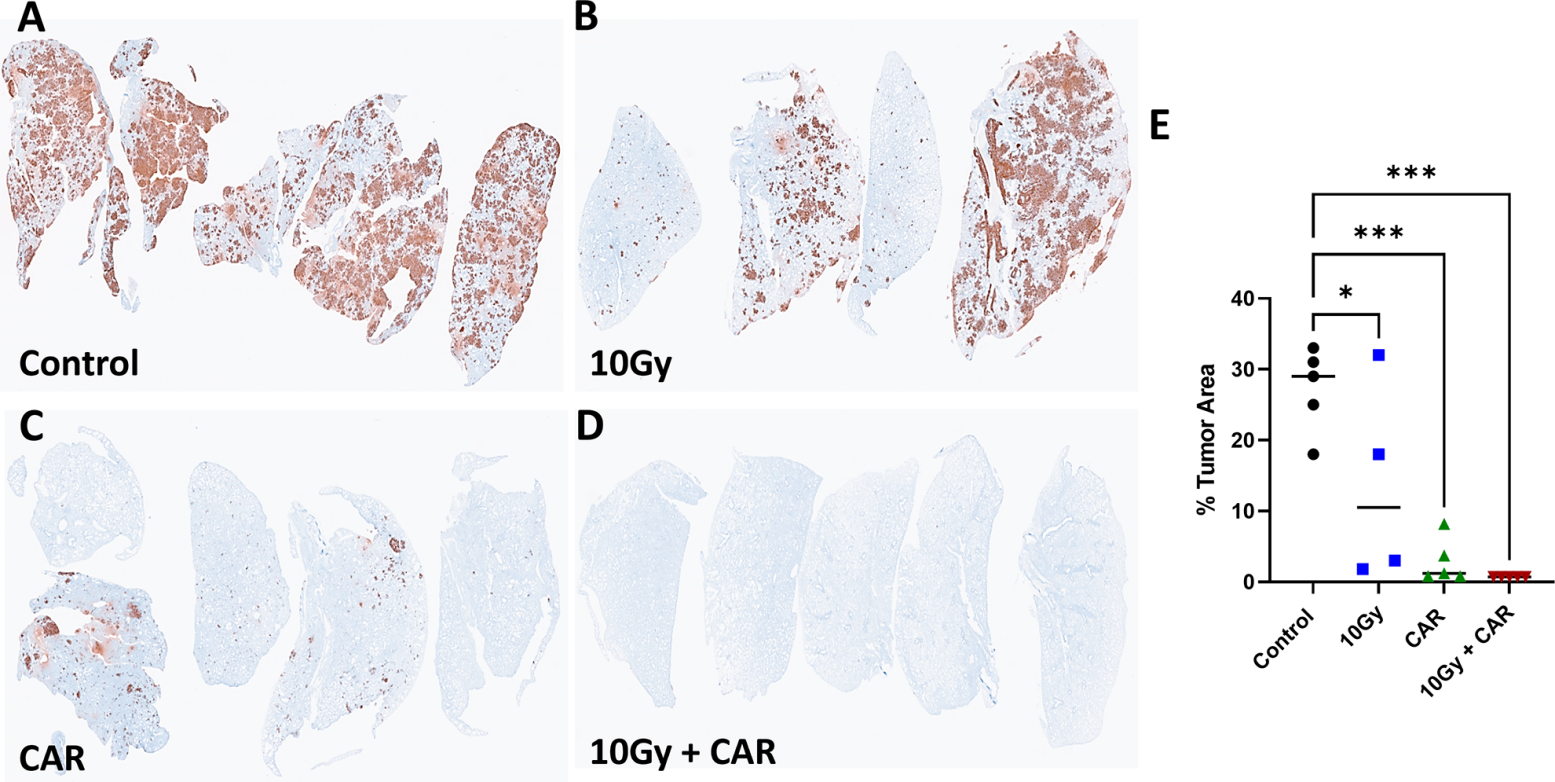
Immunohistochemistry Staining of MDA-MB231CEA-Luc Tumor Metastasis to the Lungs. At termination, one lung lobe from each mouse was collected and stained to detect Luc+ tumor cells **(A-D)**. **(E)** Quantification of Luc+ areas (average of 5 areas for each lobe) revealed a significant difference between control, single CAR-T treatment, and combination treatment groups (p < 0.001). Each group consisted of 4-5 mice. (Statistical analysis was performed using one-way ANOVA and Sidak’s multiple comparison test *p < 0.05, ***p < 0.001).

Triple-negative breast cancer is known to metastasize to the lungs, so we performed IHC analysis of lung tissue to detect luciferase-positive tumor cells. Massive metastases were found in the control group, while slightly fewer metastases were detected in the mice treated with 10 Gy IGRT alone. Interestingly, relatively few metastases were observed in the CAR-T only treated mice, while the combination of IGRT and scFab anti-CEA CAR-T therapy almost eliminated lung metastases (**Figures 6A–D**). Luciferase staining of the lung lobes indicates metastatic lesions, and their quantification showed a significant bigger tumor aera in the lungs in the control group compared to the combination treatment group (**Figure 6E**). A similar pattern was observed when the experiment was repeated with a different CAR-T cell donor and included the scFab 4-1BBzeta variant (**Supplementary Figure S4**). These data demonstrate that the combination of IGRT and anti-CEA CAR-T therapy elicits a strong antitumor response against both the primary tumor and distant metastatic spread to the lungs.

### Kinetic tracking CAR-T cells tumor infiltration *in vivo*

**Figure 7:**
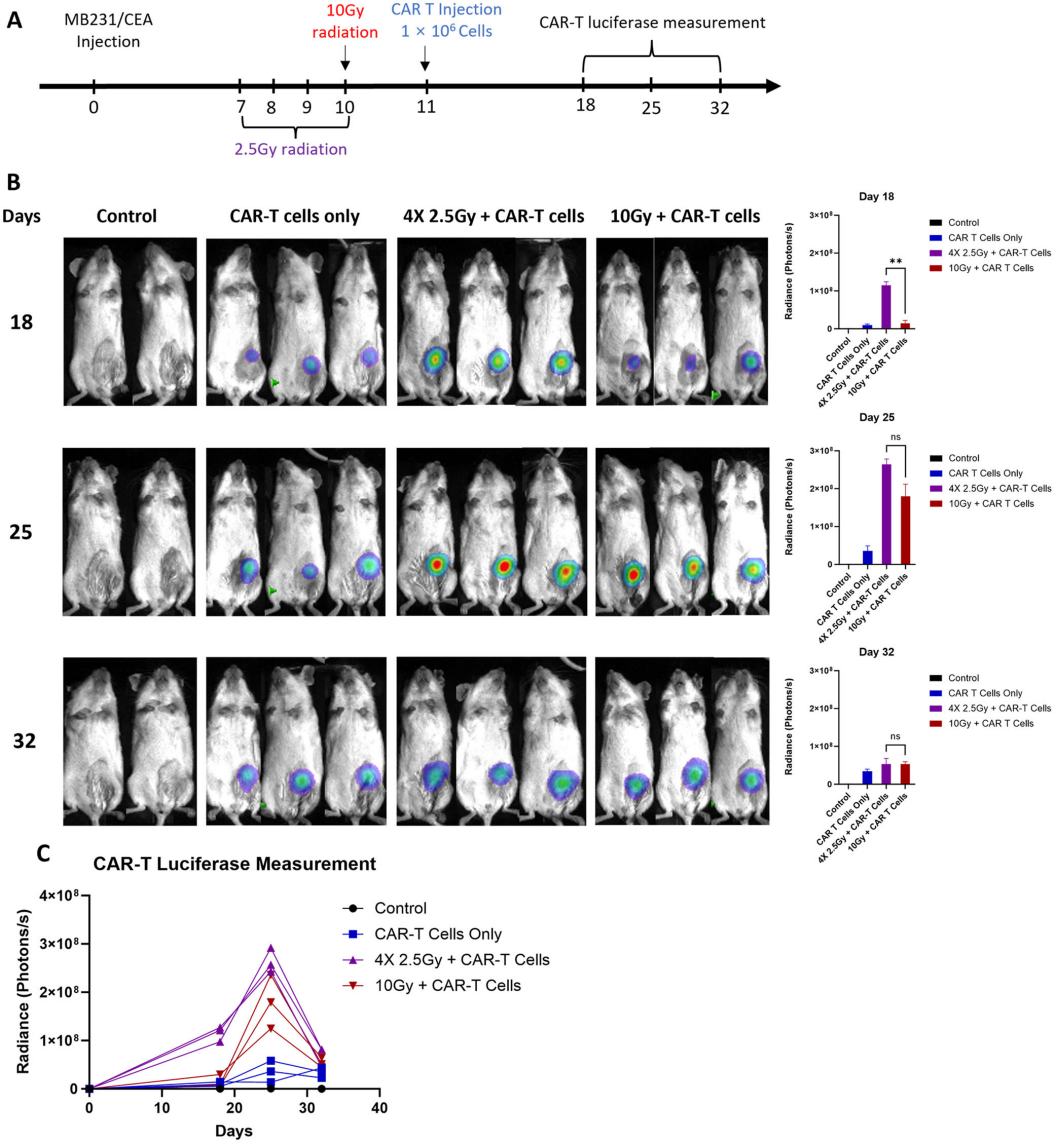
*In vivo* analysis of CAR-T cell tracking kinetics. **(A)** the treatment schedule schematic, **(B)** representative bioluminescence scans for CAR-T cell luciferase activity, and **(C)** the graphical quantification of luciferase in photons per second across the treatment groups. The untreated control group included 2 mice, while the other groups consisted of 3-5 mice each. (Statistical analysis was performed using two-way ANOVA and Sidak’s multiple comparison test **p < 0.01, ns - not significant).

Monitoring CAR-T cell tumor infiltration and proliferation *in vivo* provides an important insight into kinetics and effectiveness of the therapy. In this study, we co-transduced T cells with two lentiviral vectors expressing anti-CEA scFab CAR and GFP-Luciferase, respectively, to examine the kinetics of CAR T infiltration, preceded by 4 x daily 2.5 Gy IGRT to extend the TME effects over a longer period, as previous described (29). The treatment schedule is outlined in (**Figure 7A**) and production of double-positive (CD19 and GFP-Luciferase) CAR T-cells is shown in (**Supplementary Figure S5**). One day following IGRT, the mice were intravenously injected with 1x10^6^ anti-CEA scFab CAR-T cells expressing luciferase. CAR-T cells were tracked in NSG mice bearing orthotopic MDA-MB231/CEA tumors (without luciferase) on days 18, 25 and 32 after treatment using bioluminescence. The CAR-T cell bioluminescence was low for all groups at day 18, peaked on day 25, and returned to low values by day 32 that corresponded with objective eradication of the tumors in both combination groups (**Figures 7B, C**). Interestingly, the bioluminescence data of day 18 showed significantly higher signal in fractionation IGRT+CAR-T group suggesting more efficient TME remodeling. The results for the CAR T only group have consistently low luminescent values demonstrating that few CAR T cells enter the tumor, take a week to expand to their maximum luminescent signal, and thereafter decline. Similar kinetic profiles are seen for the combination therapy groups with highest overall values for the combination therapy. We conclude (a) that the number of CAR T cells arriving at the tumor determine the degree of expansion, explaining the similar kinetics for all three groups, and (b) that IGRT increases that number, explaining the magnitude of the expansion in terms of luminescent signal. These findings underscore the importance of pretreatment of the tumor with IGRT and appropriate timing of the subsequent CAR T therapy.

## Discussion

CAR-T adoptive immunotherapy has been one of the most successful cellular therapies to date, particularly for hematological malignancies. However, the immunosuppressive TME of solid tumors presents challenges that require strategies to enhance the homing and expansion of CAR-T cells within the TME. Among the approaches to improve CAR-T cell infiltration into solid tumors, low dose radiotherapy, especially low dose image guide radiotherapy stands out (32, 33).

Selecting the best tumor antigen for T cell mediated immunotherapy is critical both for efficacy and reducing off target toxicity. CEA was chosen as a tumor antigen due to its broad application in tumor detection, diagnosis, prognosis, and treatment monitoring, and its association with metastatic breast tumors (20). The humanized anti-CEA antibody M5A has shown efficacy with no toxicity in several clinical trials to date (19, 34). A preclinical study with anti-CEA CAR-T cells from another group targeting the A3B3 domain of CEA, also showed specific targeting with no off-target toxicity in CEA transgenic mice (23). Our *in vitro* results with our anti-CEA scFab CAR-T cells exhibited good transduction efficiency, activation, and proliferation (**Figure 2**). The cellular cytotoxicity of scFab CAR against triple-negative breast cancer cells transfected with human CEA (MB231/CEA) vs CEA negative cells showed a high specificity and excellent E:T ratios.

In our initial study using syngeneic mouse anti-CEA CAR-T cells, we used a scFv construct that showed significant activity when combined with IL-2 antibody fusion immunocytokine (22). Despite its efficacy against a syngeneic solid tumor mouse model, the humanized anti-CEA scFv construct proved unstable in xenograft CAR-T therapy. Consequently, we designed and tested a scFab CAR-T cell, which includes one constant and one variable domain from the heavy and light chain of the humanized anti-CEA antibody M5A (21). The concept of using scFab fragments stems from numerous reports examining the structure and therapeutic advantages of antigen-specific fragments of antibodies produced through recombinant processes (35). scFab-CAR-T cells were first tested by Duan and colleagues, who reported that novel Fab-CAR-T cells demonstrated heightened recognition of tumor antigens in human thyroid cancer cells and extended the lifespan of CAR-engineered T cells, generating a durable antitumor response (26).

In our studies we tested scFab CAR-T in two variants of costimulatory signaling domains CD28 or 4-1BB. Previous studies showed that CD28-based CAR-T cells usually resulted in a more robust proliferative response and effector memory T cells, whereas 4-1BB co-stimulation induced a progressive response and with enhanced persistence and central memory differentiation (36). Moreover, the work by Starr et al. (37) showed improved specificity, persistence, and efficacy of 4-1BB–based IL13-ligand CARs when compared to CD28 format (37). Nonetheless, the selection of the co-stimulatory domain remains controversial and may be influenced by the structure of CAR molecules and the histopathology of the target diseases. However, our *in vitro* and *in vivo* studies have proven similar anti-tumor effects for both scFab CAR-T formats.

As expected, treatment of orthotopic TNBC cancer-bearing mice with our anti-CEA scFab CAR-T cells alone did not result in significant infiltration of these cells into the tumor or tumor inhibition. However, combination treatment with IGRT (single low dose of 10 Gy) resulted in synergistic activity, leading to stronger tumor inhibition compared to individual treatments. A repeat of the experiment with a different donor T-cell also showed similar tumor growth inhibition. For CAR-T cells to effectively kill tumor cells, they must sufficiently infiltrate the hostile tumor microenvironment (38). The combination of anti-CEA scFab CAR-T cells with IGRT increased CAR-T cell infiltration into the tumor as shown by immunostaining and luciferase labeling of the CAR T cells. A similar observation was also reported by Quach and colleagues, who found that tumor-targeted radiation prior to systemic administration of CAR-T cells substantially improved CAR-T cell therapy efficacy and infiltration in solid tumors (39). Akhavan et al. (40) investigated the effects of stereotactic radiation therapy at doses of 5, 10, and 20 Gy on the TME in a GBM murine tumor model. They found that a conditioning dose of 10 Gy was particularly effective in stimulating cells to enhance tumor growth kinetics and induce gene expression changes that support the combination with CAR-T cell immunotherapy (40). Additionally, other groups have reported that administering a sub-cytotoxic radiotherapy dose of 0.5 – 2Gy followed by CAR-T cells infusion have increased the regulation of death receptor molecules to enhance CAR-T cell efficacy (41).

An alternative approach by Cao and colleagues found that combining microwave ablation radiation therapy with AXL-CAR-T cells resulted in superior antitumor efficacy. Their findings suggest that tumor guided radiation enhances the activation, infiltration, persistence, and tumor-suppressive properties of AXL-CAR-T cells in non-small cell lung cancer patient-derived xenograft tumors via tumor microenvironment (TME) remodeling (42). Treatment of antigen-heterogenous pancreatic cancer with low-dose radiation therapy and CAR-T cells demonstrated that localized radiation can sensitize antigen-negative tumor cells, which would otherwise evade CAR recognition, to be effectively eliminated by CAR-T cell killing (43). IGRT prior to immunotherapy can cause tumor TME remodeling and depletion of some immunosuppressive cells, enhancing CAR-T cell migration to the tumor site. Thus, the combination therapy induced significant tumor suppression without observed toxicity in humanized immunocompetent mice.

However, the risk of induction of metastatic spread to distant organs caused by primary tumor irradiation has been less studied. Bouchard et al. (44) investigated the impact of radiation on the mammary glands, focusing on the invasiveness of breast cancer cells that survive radiation treatment. Their findings revealed a significant increase in breast tumor cell migration from the primary tumor compared to non-irradiated controls. This was associated with elevated expression of pro-migratory and pro-inflammatory molecules such as IL-6, cyclooxygenase-2, membrane type 1 metalloprotease, phospholipase A2, and transforming growth factor-β (TGF-β), which likely facilitated the migration of cancer cells, increased circulating tumor cells, and metastasis to the lungs. Supporting this, Biswas et al. (45) showed that increased secretion of TGF-β by stromal cells post-irradiation promoted lung metastases in an orthotopic mammary tumor model. Similarly, irradiation of hepatoma cells has been linked to the secretion of tumor necrosis factor-alpha (TNF-α), IL-6, VEGF, epidermal growth factor (EGF), MMP2, and MMP9, all of which enhance tumor invasion (46). Additionally, radiation has been shown to favor cancer cell migration at the expense of primary tumor growth in a glioblastoma rat model. Brain irradiation before primary tumor implantation promoted the infiltration of cancer cells into distant organs and induced a phenotypic shift in glioma cells from a proliferative to an invasive type (47).

The impact of vascular changes following IGRT is controversial. For example, Castel and Kirsch (48) reported that high-dose radiation causes endothelial cell proliferative defects, leading to increased vascular permeability and subsequent tumor cell death. However, Budach and colleagues (49) found no difference in local tumor control across various human cell lines using the high radiation dose necessary to cure 50% of tumors implanted in nude or SCID mice. Their findings suggest that stromal endothelial cells do not significantly influence tumor control by radiation, despite differences in the radiosensitivity of the mice used, supporting the theory of direct tumor killing (49). When we increased the radiation dose to 20 Gy, we observed a delayed tumor growth curve (results not shown) in the primary tumor, but this delay did not translate into long-term tumor control or elimination. Thus, it appears that low dose IGRT rather than high dose IGRT is preferable. Further improvements are possible with fractionated IGRT that affects tumor growth over a longer period. In a pilot study tracking CAR-T cell activity with luciferase after fractionated IGRT indicated the highest infiltration and expansion levels when tumors received four low daily doses of radiation. This insight highlights the need for further research to thoroughly understand the timing of CAR-T cell therapy after IGRT, with additional consideration of the radiation dosing schedules and possibility of multiple CAR-T cells treatments.

Importantly, our study demonstrated that the combination of IGRT and anti-CEA scFab CAR-T therapy elicited not only a strong antitumor response, but also prevention of metastatic spread to the lungs. As a mechanism, we suggest that low dose IGRT at the primary tumor enhanced CAR-T cell infiltration and expansion in the primary tumor, allowing sufficient persistence and increased CAR T trafficking to distant metastatic sites as they developed over time.

This study was limited to CAR-T therapy in immunocompromised animals, which restricts the ability to assess potential toxicity, an issue that we and other groups have previously shown in CEA transgenic mice treated with murine CAR T therapy without off-target effects (22, 23). Another limitation is that a detailed cytokine panel analysis at different time points was not performed, leaving open the question of involvement of other activation markers aside from IFN-γ and granzyme B. The exhaustion markers analysis was limited to PD-1 and TIM3. However, the emergence of newer markers like Tox and CD39 in the measurement of T-cell exhaustion may better explain the observations of tumor escape (50, 51). These aspects will be addressed in future experiments. Nevertheless, in our study the majority of the CD8+ CAR-T cells were PD-1 negative and expressed high levels of IFN-γ, both markers of effective CAR T therapy in clinical studies.

In summary, this study highlights the potential of anti-CEA scFab CAR-T cells as a promising therapeutic approach in combination with low dose IGRT. Building on our observations of the synergistic activity of anti-CEA scFab CAR-T cells with image-guided radiotherapy represents a novel therapeutic option that warrants clinical evaluation in solid tumor patients with metastases.

## Data Availability

The original contributions presented in the study are included in the article/**Supplementary Material**. Further inquiries can be directed to the corresponding author.
