# Supplementary material for: Targeting CEA in metastatic triple negative breast cancer with image-guided radiation followed by Fab-mediated chimeric antigen receptor (CAR) T-cell therapy
Source: Front Immunol. 2024 Dec 20;15:1499471. doi: 10.3389/fimmu.2024.1499471 (PMC11695362; doi:10.3389/fimmu.2024.1499471)
Supplement: Supplementary file 1 [file DataSheet1.pdf]

Supplementary Information

Targeting CEA in metastatic triple negative breast cancer with image-guided radiation followed by Fab-mediated chimeric antigen receptor (CAR) T-cell therapy.

Eric Aniogo[a], Maciej Kujawski[a], Dennis Awuah[b], Seung E. Cha[a], Ruby Espinosa[b], Susanta Hui[c], Hemendra Ghimire[c], Paul J. Yazaki[a], Christine E. Brown[d], Xiuli Wang[b] and John E. Shively[a].
[a]Department of Immunology and Theranostics, City of Hope, Duarte, California, USA.
[b]T-Cell Therapeutic Laboratory, City of Hope, Duarte, California, USA.
[c]Department of Radiation Oncology, City of Hope, Duarte, California, USA.
[d]Department of Hematology & Hematopoietic Cell Transplantation, City of Hope Beckman Research Institute and Medical Center, Duarte, California, USA.

Supplementary information is included.

- **4 Supplementary Figures and legends**

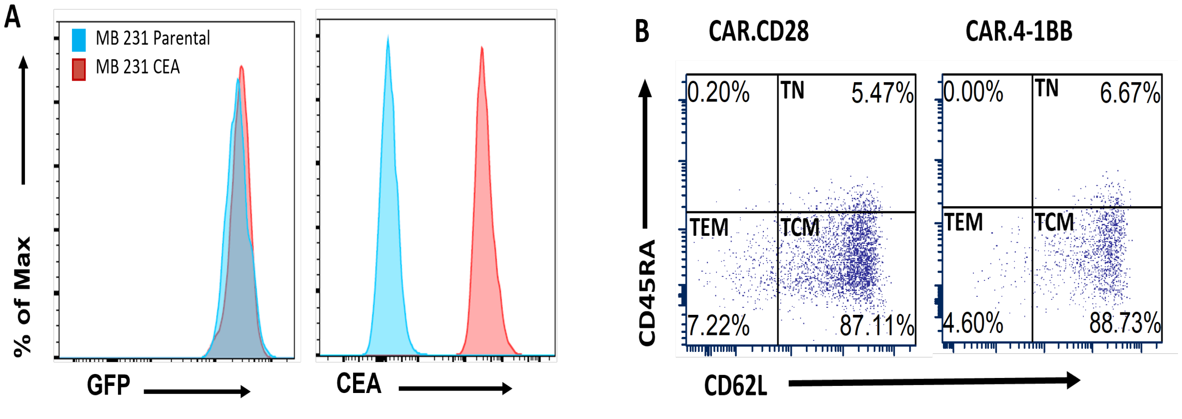

Supplementary Figure S1. (A) Expression of CEA in parental and MB231/CEA cells. (B) Characterization of donor T cells, showing the naïve/memory T cells populations used for CD28 and 41BB CAR T cell development.

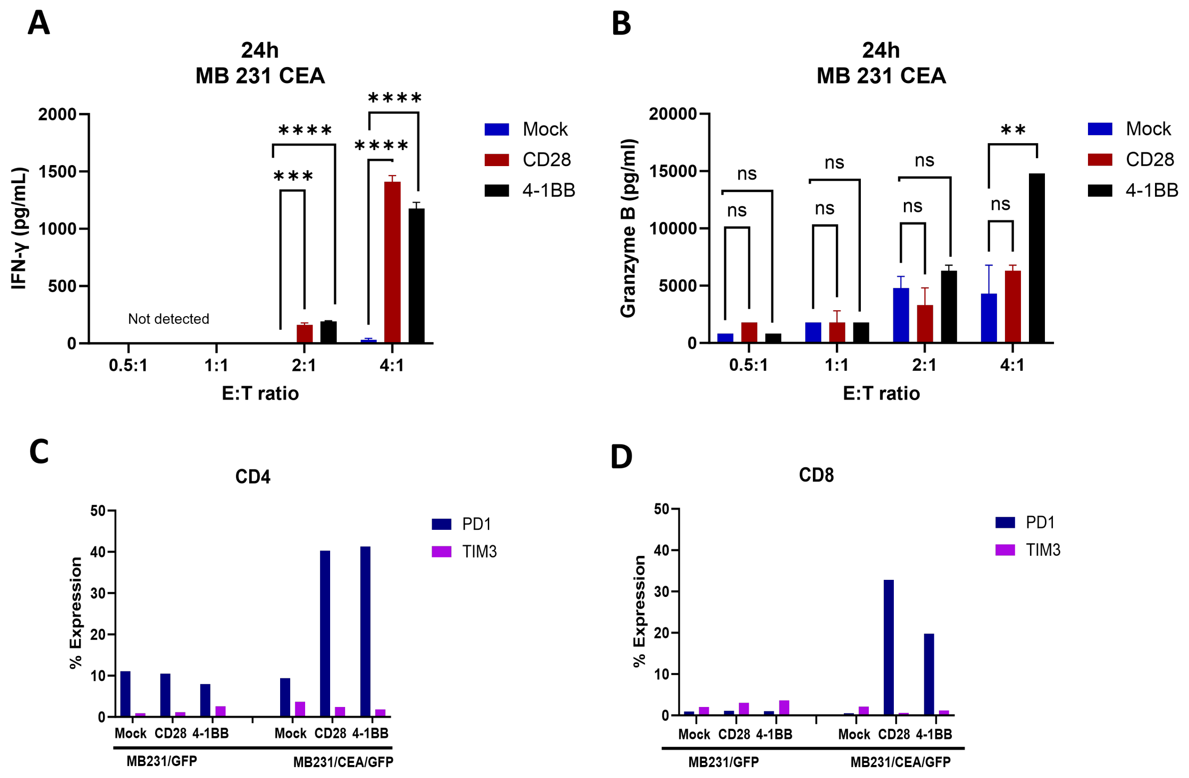

Supplementary Figure S2. (A) and (B) display ELISA analysis of IFN-γ and Granzyme B respectively of mock, anti-CEA CD28-CAR-T, and 41BB-CAR-T for different E:T ratio groups measured using the CLARIOstar instrument. (C) and (D) displays the percentage expression of PD-1 and TIM-3 exhaustion markers on CD4 and CD8 T cell subpopulations in both CEA-negative and CEA-positive MDA-MB231 cells.

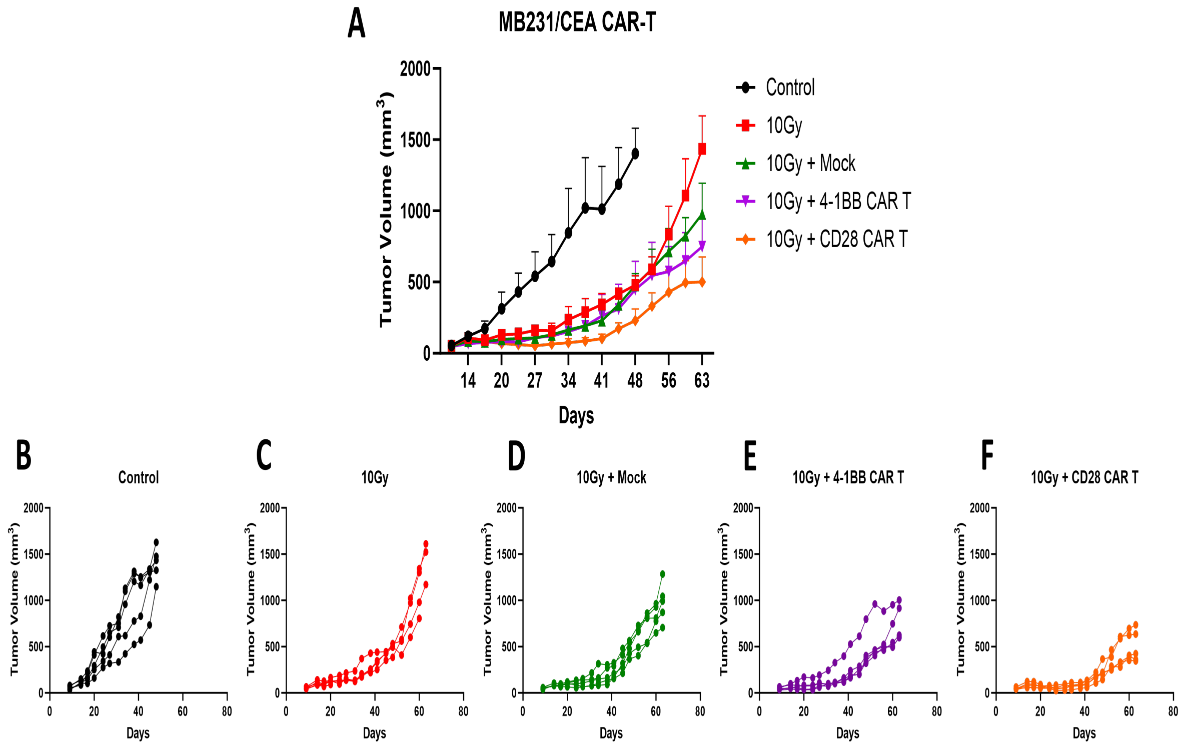

Supplementary Figure S3. (A) Tumor growth curves for MDA-MB-231CEA-Luc, with 4-5 mice per group, each receiving T cells from different donor T cells. (B) Individual tumor growth curve(s) of mice for the indicated treatment groups.

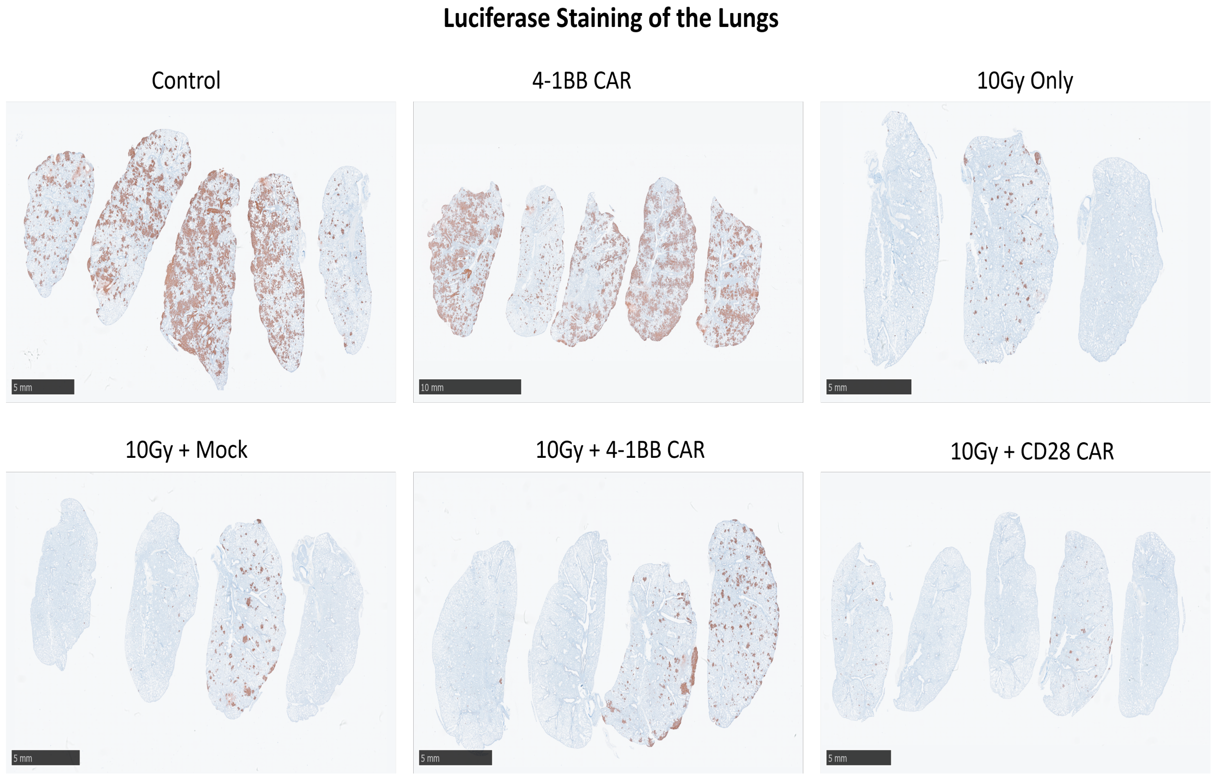

Supplementary Figure S4. Immunohistochemistry Staining of a repeat experiment using a different CAR-T donor, showing MDA-MB231/CEA-Luc tumor Metastasis to the Lungs. The images represent the IHC staining of one lung lobe per mouse, performed at the experimental endpoint to detect Luc+ tumor cells. Each group consists of 3-5 mice.

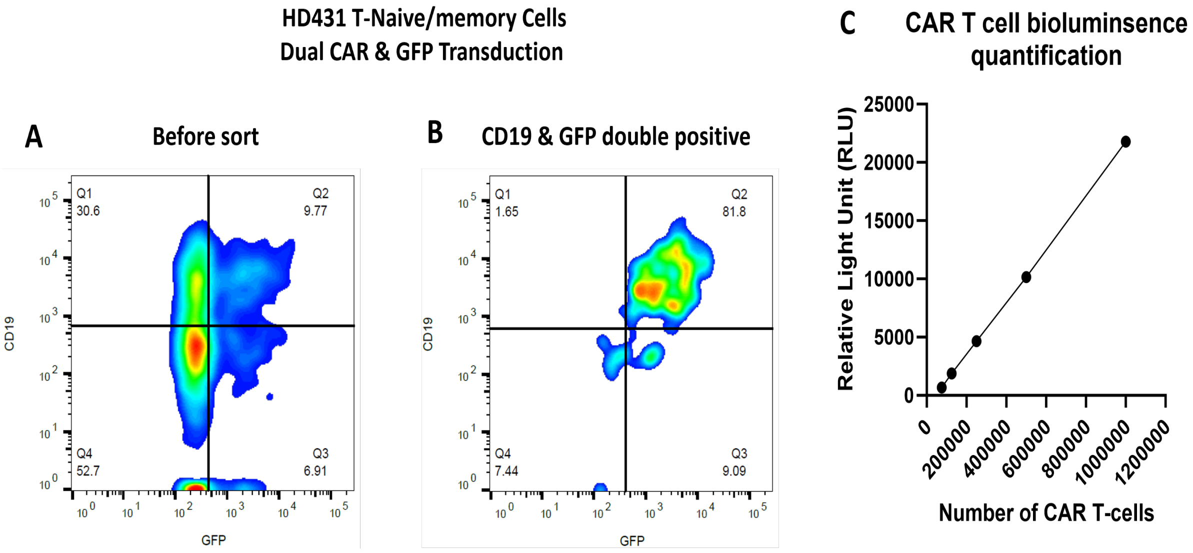

Supplementary Figure S5. (A) and (B) display flow cytometry data showing the expression of CD19+ AND GFP+ cells before (A) and after (B) sorting, with percentages of positive cells shown. (C) shows the linear correlation between the sorted CAR T cells and luciferase bioluminescence after exposure to D-luciferin, measured using the CLARIOstar instrument.
